# Breast cancer metastasis: Is it a matter of OMICS and proper ex-vivo models?

**DOI:** 10.1016/j.csbj.2022.07.044

**Published:** 2022-07-28

**Authors:** Mario Cioce, Andrea Sacconi, Sara Donzelli, Claudia Bonomo, Letizia Perracchio, Mariantonia Carosi, Stefano Telera, Vito Michele Fazio, Claudio Botti, Sabrina Strano, Giovanni Blandino

**Affiliations:** a Laboratory of Molecular Medicine and Biotechnology, University Campus Bio-Medico of Rome, Rome, Italy; b Institute of Translational Pharmacology, National Research Council of Italy (CNR), Rome, Italy; c Clinical Trial Center, Biostatistics and Bioinformatics Unit, IRCCS Regina Elena National Cancer Institute, Rome, Italy; d Translational Oncology Research Unit, IRCCS Regina Elena National Cancer Institute, Rome, Italy; e Department of Pathology, IRCCS Regina Elena National Cancer Institute, Rome, Italy; f Neurosurgery Unit, IRCCS Regina Elena National Cancer Institute, Rome, Italy; g Laboratory of Oncology, Fondazione IRCCS Casa Sollievo della Sofferenza, 71013 San Giovanni Rotondo, Italy; h Breast Surgery Unit, IRCCS Regina Elena National Cancer Institute, Rome, Italy; i SAFU Unit, IRCCS Regina Elena National Cancer Institute, Rome, Italy

**Keywords:** Metastatic breast cancer, Organoids, PDTO, Genomic, Proteomic, multi-OMICS

## Abstract

Genomics has greatly increased the understanding of the study of breast cancer (BC) and has shaped the concept of intra-tumor heterogeneity, currently recognized as a propelling force for cancer progression. In this context, knowledge and understanding of metastatic breast cancer (mBC) has somehow lagged behind that of primary breast cancer. This may be explained by the relative scarcity of matched mBC samples, however it is possible that the mutation spectrum obtained from primary BC does not capture the full complexity of the metastatic disease. Here, we provide a few examples supporting this possibility, from public databases. We evoke the need to perform an integrated multi-OMICS characterization of mBC, to obtain a broad understanding of this complex disease, whose evolution cannot be explained solely by genomics. Pertinent to this, we suggest that rather an infrequent use of Patient-Derived –Tumor-Organoids (PDTOs) may be influenced by assuming that the metastatic conditions of PDTOs growth (mPDTOs) should be similar to those of the tissue of origin. We challenge this view by suggesting that the use of “target-organ inspired” growth conditions for mPDTOs, may better fit the emerging knowledge of metastatic disease. Thus, the integrated use of multi-OMICS and of clinically relevant mPDTOs may allow a further understanding of such disease and foster therapeutically relevant advances. We believe that our points may be valid for other solid cancers.

## 1 Introduction

Metastatic progression is the primary cause of cancer mortality, thus there is no exception to metastatic breast cancer (mBC), as more than 90 % of the cancer-related deaths are a result of metastatic [1], [2]. Considering histology and BC subtypes, 30–60 % of breast cancer patients have bone metastases, 21–32 % with lung metastases, 15–32 % with liver metastases and 4–10 % with brain metastases [3]. Metastatic disease poses a significant therapeutic challenge, with unpredictable inter- and intra-patient variability becoming a major obstacle to therapeutic intervention. Interrogating BC metastases is crucial to opening new therapeutic pathways and fulfilling unmet patient stratification needs, for diagnostic and prognostic purposes.

There is a significant lack of information on metastases, resulting in knowledge regarding metastatic disease lagging behind that available on primary tumors. As a matter of fact, while the genomic landscape of primary breast cancer has been extensively analyzed in over 2000 patients [4], analogous data for metastatic breast cancer are much less represented. Three recent studies [5], [6], [7] have sequenced about 1500 breast cancer metastases: however, the studies using paired primary tumors vs metastases from the same patient only include one fifth of those samples [5]. Furthermore, brain metastases are under-represented. Retrieving samples of brain metastases may be affected by practice bias, partially due to the increasing use of stereotactic radiosurgery and the unfeasibility of longitudinal tissue sampling [8].

**Fig. 1:**
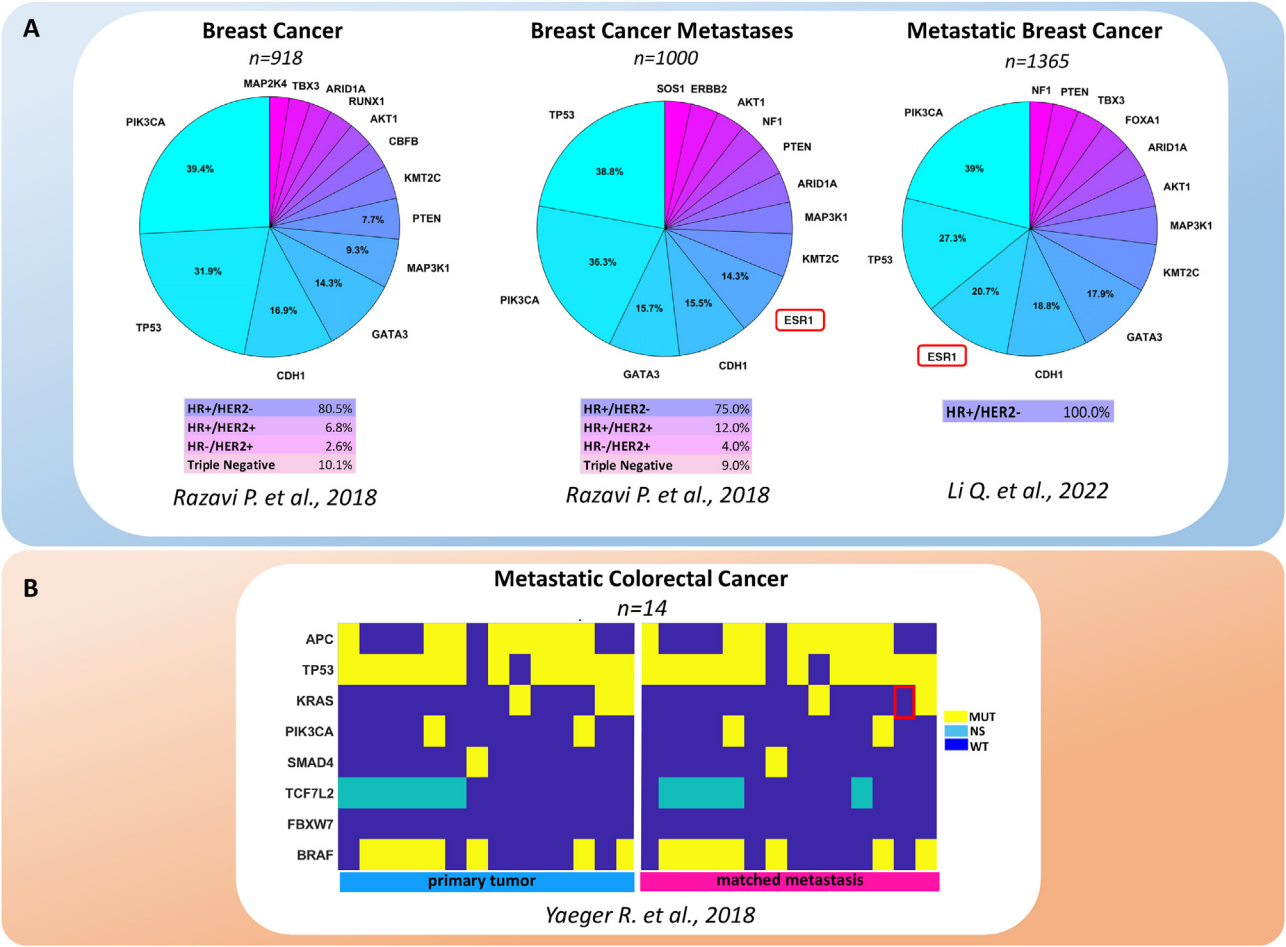
**Genomic alterations in metastatic breast and colorectal cancer.** A. Frequency of the top 13 mutated genes in Breast Cancer (n = 918), Breast Cancer Metastases (n = 1000) and in Metastatic Breast Cancer (n = 1365). Data were retrieved from the Breast Cancer MSK databases [12], [16]. B. **Genomic alteration in colorectal cancer**. The top 8 genomic alterations in Matched Primary and Metastatic colorectal cancer lesions (n = 14). Data derived from the Metastatic Colorectal Cancer MSK database [17], n = 1134 total samples. MUT: mutated; NS: not sequenced; WT: wild type.

Undoubtedly, genomics has contributed a significant amount of knowledge on mBC in recent years. For example, some genes have been identified as preferentially mutated in breast cancer metastases, when collectively considering all the studies: TP53[9]; ESR1 [5], [10], [11]; ERBB2 [12]; JAK2 [13]; NF1 [5], [12]; PALB2 [11], [14]; STAT3[13] TSC1/2 [14]; KMT2D, KTM2C [6], [7] and also ERBB3, FBXW7, GATA1, KRAS, MEN1, NF1 [5]. Moreover, when the clonal composition of the primary tumors and their paired metastases were evaluated, there was a general increase in metastatic samples. This phenomenon was prominent in HER2-expressing tumors, involving ESR1, SMAD4, RB1, ERBB2 and LRP1B [5]. Along the same line, ESR1 mutations were enriched in liver metastases of the hormone receptor (HR)+/HER2pos breast cancers. FOXA1 mutations and RHOA mutations were more represented in liver metastasis and ovarian metastasis of lobular breast cancer, respectively [7], [12], [15]. Despite this important information, without understating the translational potential of these findings, it is possible that the available repertoire of mutated genes in mBC may not fully explain the heterogeneity and clinical behavior of metastatic lesions. For example, when searching for mutations associated with metastatic proclivity of primary breast cancer, we found that the mutation spectrum was similar between primary breast cancer and breast cancer metastases, in the MSK database [12], [16]. We only found a slight increase in the frequency of ESR1 mutations in metastatic samples (Fig. 1A). Even when comparing frank metastatic lesions with matched primary cancers in other types of tumors, such as colorectal cancer [17], the difference between the primary tumor and metastatic material appeared to be inadequate to explain the heterogeneity in disease progression (Fig. 1B). This indicates that the actual potential to identify pro-metastatic determinants of breast cancer progression is quite limited and that a broader and deeper application of next generation sequencing (NGS) and single cell NGS are yet to be achieved.

As for the abovementioned, the development of targeting agents in the metastatic setting has lagged behind that of the same agents against primary tumors. Genomics-driven prediction of efficacy has provided therapeutic results in primary tumors, but much less in metastases. A concurrent problem is that less than 20 % of patients are eligible for genomics-driven drugs and less than half of the treated ones gain clinical benefit [18]. It is likely to expect that even a smaller fraction of patients carrying metastatic disease would benefit from those drugs.

Still, the important advances that have taken place in genomic-driven drug discovery, even in metastatic settings is worth mentioning. This is the case for vemurafenib in metastatic melanoma expressing BRAFV600 [19] and for trastuzumab in HER2positive metastatic BC. Furthermore, alpelisib, a PI3Kalpha inhibitor [20], is currently being explored in phase 1b, specifically in HER2pos metastatic breast cancer, within combined and chemotherapy sparing settings [21]. This is based on the evidence involving PIK3A mutations in resistance to anti-HER2 agents [22], [23]. Importantly, the contribution of genomic findings towards increasing clinical benefit, for example when excluding anti-EGFR therapies in advanced/metastatic colorectal cancer patients bearing KRAS mutations [24], should be acknowledged.

Given the possibility that additional mutations and pathway disturbances have yet to be discovered, it is well known that the very heterogeneous clinical response of metastases to the therapy, even within the same patient, is an unsolved therapeutic challenge. This scenario is made more complex by the evidence, in other cancer settings, that the timing of genomic alterations during cancer progression may be uncoupled from histological progression [25]. This latter observation pairs with emerging clues that the functional effect of mutations and genomic alterations is strongly influenced by the status and identity of the carrying cells each time of the history of the disease [26], [27]. Altogether, this suggests that sequencing alone may not suffice to explain metastatic disease.

## 2 There is need for a “beyond genomics” approach

Recent work has shown that metastatic lesions with very similar gene expression profiles were indeed very different when studied from a multi-OMICS perspective. In fact, when assessing the proteomes, genomes, and metabolomes of 16 resected brain metastases concomitantly, Dr. Su and colleagues show how multi-OMICS may greatly enhance and integrate prognostic information deriving from the gene expression profile [28]. The number of reports suggesting the usefulness of multi-OMICS analysis increasing the specificity and sensitivity of biomarker discovery is growing daily. Similarly, the potential for applying multi-OMICS to single cell analysis for pathway discovery, like in pancreatic cancer [29], is emerging. Multi-OMICS may help in understanding the heterogeneous clinical response of the metastases (a real therapeutic dilemma for oncologists) and the limited clinical usefulness of employing “single assay” approaches. An example of how a multi-OMICS approach could be informative comes from the study evaluating the EGFR post-translational modifications. For example, assessing the phosphorylation of EGFR at T654 was shown to highly correlate with the metastatic potential of BC and may certainly refine and increase the clinical utility of detecting matched DNA mutation in patients. Thus, even in the presence of common genomics and transcriptomics features, the EGFR posttranslational status of the receptor may represent a clinical determinant towards using EGFR TKI in metastatic BC settings [30].

The reported work and our own experience converge on one concept: while malignant transformation and tumor progression critically depend on somatic mutations and their study has been instrumental in unveiling intra-tumor heterogeneity (ITH) in primary tumors, metastasis may be crucially driven by posttranscriptional and posttranslational changes. The relative weight of those changes may be pivotal for establishing the identity of the metastatic lesion. This also calls for reinterpreting the dynamic concept of epithelial to mesenchymal (EMT) and mesenchymal to epithelial (MET) transition [31], [32], [33], [34] as driving forces behind tumor dissemination and growth of metastases in the target organ, respectively. A multi-OMICS analysis may indeed capture the complex picture of cell plasticity and metastasis in detail, when compared to conventional genomics studies.

## 3 Which multi-OMICS data layers should we consider?

The logical answer to this question would of course be: genome, epigenome, transcriptome, proteome, glycome and metabolome. However, we can provide some examples deriving from mainstream OMICS approaches such as metabolomics and proteomics. Different to primary breast cancers metabolomics has shown distinct traits in correlation with histopathology, higher expression of Her2 [35], [36], [37] and microenvironment composition [38], [39], much less is known about the metabolomic profile of mBC lesions. Consequently, current knowledge on the crosstalk between metabolic reprogramming and the metastatic process in breast cancer is scarce. What is accepted so far is that the metabolic plasticity correlates with high metastatic potential. In fact, metastatic cells exhibit the ability to use multiple metabolic pathways concurrently, in order to support their micro-environmental adaptive capacity [40], [41]. A few examples of this include: liver-metastatic breast cancer cells displaying an accumulation of glucose-derived lactate and a reduction in the tricarboxylic acid cycle and oxidative phosphorylation [42]. Furthermore, studies from patient-derived-xenograft models suggested that breast cancer cells that metastasized to the brain enhanced glucose oxidation, mitochondrial respiration, pentose-phosphate-pathway (PPP) and increased glutathione synthesis, when compared to the cells derived from the originating tumor [43]. Additionally, brain metastases from human breast cancer patients expressed higher levels of fructose bisphosphate and glycogen than the corresponding primary tumors. *In vitro* studies showed that this supported the cancer cells ability to survive and proliferate independent of glucose, thereby employing non-oxidative PPP for purine synthesis [44]. Overall, the degree of metabolic heterogeneity seems generally not to increase or even decrease when comparing primary tumors with their metastatic counterparts [45], possibly reflecting the result of positive selection within the tumor microenvironment. This is partially unlike what genomics has shown on the increased clonality of the metastasis as compared to the primary tumors, thus warrants investigation.

**Proteomics.** Micro-proteomics based “on tissue micro digestion” may reveal differentially expressed proteins in breast metastasis, but in a topographically informative way. By using such a clonal proteomics approach, it has been shown that about half of the proteins differentially expressed between breast primary tumors and metastases were not redundant to TCGA and partly belonged to understudied, druggable pathways with clinical relevance for breast cancer [46]. This again underlies the utility of complementary approaches to genomics towards understanding the biology of metastatic progression. Another important point to note is that proteomes (and metabolomes) may be affected by factors, such as nutrient deprivation or hypoxia, influenced by the genomics status only to a limited extent. Altogether, this strengthens the idea that proteomic and metabolomic heterogeneity may not “simply” stem from genetic heterogeneity.

## 4 Challenges: Integrating big data collections.

There is of course a great challenge waiting for multi-OMICS right around the corner, that is, systematically integrating such heterogeneous information to accurately define the regulatory networks that are critical for metastasis. The progress that bio-simulation approaches have made, based on the possibility of using multi-OMICS data to create patient personalized models [47] and predict treatment readouts is also worth mentioning [48]. Although somewhat in its infancy, bio-simulation looks like a promising tool for the future. To summarize this first part of the mini-review, evaluating genomic alterations by means of sequencing methods has allowed us to recognize emerging clonal cancer cell subpopulations, in addition to providing a few targets whose therapeutic exploitation appears promising. However, it is now clear that dynamic transcriptional, post-transcriptional, translational and metabolic events clearly shape the adaptive potential of metastases and fuel the clinical heterogeneity of metastatic disease. Thus, the roots for such heterogeneity and hence the clinical response, lies within additional layers of data and where both metabolomic and proteomic studies have started showing a non-redundant heterogeneity integrating the genomic one. Furthermore, single OMICS approaches such as navigating the genome for cancer mutations and identifying altered epigenetics or the differential expression of mRNA and proteins are not enough to unravel such a complex setting. All this indicates that multi-OMICS may be the way to go when approaching clonal theranostics.

## 5 Do we have the right models for mBC?

Besides the current or expected progress of multi-OMICS studies, there is a need for experimental models, amenable to predictive drug testing, for breast cancer metastases. Patient-derived tumor organoids (PDTOs) were shown to retain the histological complexity and genetic heterogeneity of parental tumors which may represent an interesting solution to this issue. PDTOs may be relevant for validating treatment strategies at the level of individual patients [49], [50]. On the other hand, PDTOs lose components of the *in vivo* microenvironment and may develop niche-independency during passaging [51], [52]. Possibly more important for this review, generating organoids from metastatic material does not appear to be a simple task.

In fact, growing organoids from breast metastases has not been a focus of organoid –based research in recent years. To the best of our knowledge, there are no studies specifically aimed at setting up 3D cultures from metastatic spreads of breast cancer, despite insightful examples deriving from other cancer settings, such as colorectal cancer [53]. For example, in a seminal study by Sachs and colleagues, only 13/175 samples collected were from breast metastases, with the remaining part represented by primary tumors [54]. The paucity of studies may be due to, as mentioned before, practice bias, since in clinical settings the removal of brain and spine metastases is not a generalized approach. One other possibility is that there may be intrinsic technical difficulties in obtaining 3D cultures from metastatic material. We will try to break down this latter argument below.

The composition of an organoid growing medium (OGM) is what makes the difference in yielding organoid formation from normal and diseased tissues. As such, adding tissue specific factors on top of conventional “backbone” medium, relies on a certain tissue specificity which is instrumental in successful organoid culturing. This is in-line with the evidence showing that significant differences do exist in the growth media of liver cancer-derived organoids as opposed to breast- or brain- cancer derived ones [55]. The rather consolidated view of the metastasis as a secondary localization of the primary tumor, implies that the metastatic tissue “remembers” the bio-architecture, hence the gene expression profile and mutational profile of the originating tumor. Based on this, culturing metastatic material with an OGM specific for the “tissue of origin” (e.g. primary tumor) may appear to be the logical choice. “Simple” clinical evidence depicting that primary BC can be targeted therapeutically with very different success rates when compared to mBC, is sufficient to challenge this view. Consistent with this, one- fourth to one- fifth of the metastatic BCs do exhibit hormone receptor (HR) and HER2 status discordant from the primary tumor [56]. Furthermore, subtype switching was recorded in more than one-third of mBC cases [5]. On the other hand, it has been shown that target organ site is a major determining factor of the genomic landscape of metastatic lesions [15]. Of note, while the tropism for the target organ is related to the subtype of the primary tumor [57], established metastatic lesions may undergo changes in order to adapt to the new microenvironment. Relevant to this, brain metastatic cancer cells acquire certain metabolic characteristics common to neuronal cells [58]. For example, “GABAergic” breast tumor cells have been described as being capable of surviving by converting gamma-amino-butyric acid to succinate in order to increase the citric acid cycle [59]. Thus, established metastatic tissue does not mandatorily keep the functional bio-architecture of the originating primary tumor which may be instrumental for adapting to the new microenvironment.

How such observations may apply to culturing and propagating metastatic material does pose an interesting question. In fact, if the metastatic material is “prompted” to mimic the tissue of destination, then the composition of the OGM medium should be formulated by taking into consideration the destination tissue, rather than the “originating” tissue. We are currently evaluating such an alternative approach towards growing mBC-derived PDTOs.

## 6 Summary and outlook

**Fig. 2:**
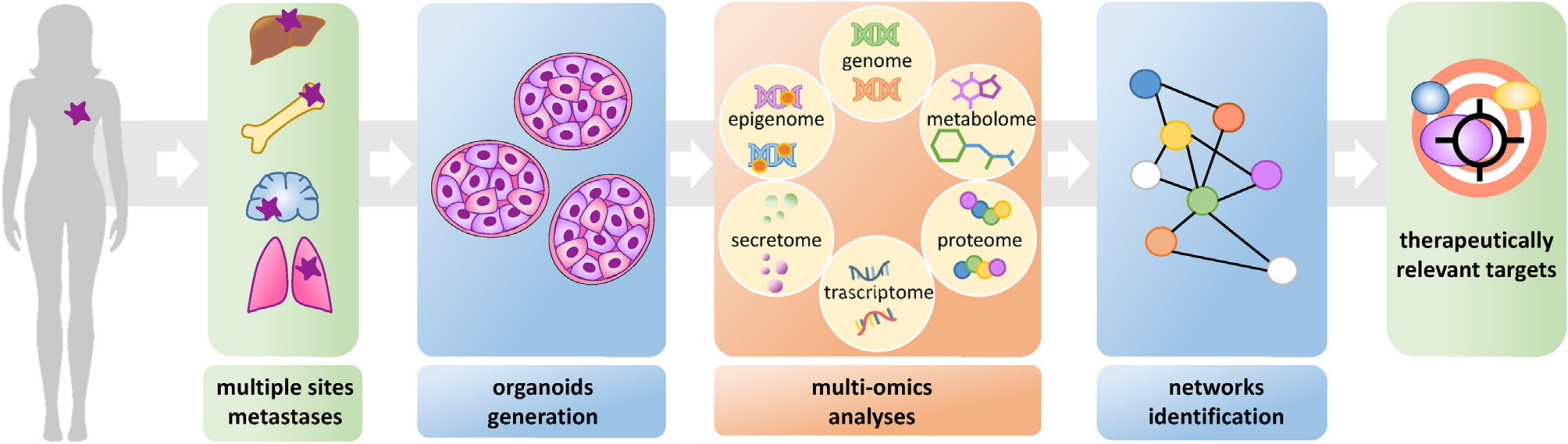
**An integrative multi-OMICS approach** directed at capturing the mBC complexity through the use of target tissue-directed culture conditions of PDTOs from mBC.

In summary, here we speculate that developing PDTO culture conditions from metastatic material and integrating multi-OMICS approaches to the study of those structures may pave the way to a therapeutically relevant scenario. This may aid in filling in current knowledge gaps and delivering clinically actionable information. Fig. 2 provides a schematic summary of the considerations reported in this mini-review (Fig. 2).

## CRediT authorship contribution statement

**Mario Cioce:** Conceptualization, Formal analysis, Writing – original draft, Writing – review & editing. **Andrea Sacconi:** Formal analysis, Writing – review & editing. **Sara Donzelli:** Writing – review & editing. **Claudia Bonomo:** Writing – review & editing. **Letizia Perracchio:** Writing – review & editing. **Mariantonia Carosi:** Writing – review & editing. **Stefano Telera:** Writing – review & editing. **Vito Michele Fazio:** Writing – review & editing. **Claudio Botti:** Writing – review & editing. **Sabrina Strano:** Conceptualization, Writing – review & editing. **Giovanni Blandino:** Conceptualization, Writing – original draft, Writing – review & editing.

## Declaration of Competing Interest

The authors declare that they have no known competing financial interests or personal relationships that could have appeared to influence the work reported in this paper.
